# Supplementary material for: AI versus human coding of NIH grant abstracts
Source: PLoS One. 2026 Oct 8;21(10):e0343857. doi: 10.1371/journal.pone.0343857 (PMC13648982; doi:10.1371/journal.pone.0343857)
Supplement: Supplementary file 1 [file pone.0343857.s002.docx]

**Q4: PRIMARY OUTCOME**

I will provide you with an abstract from an NIH funded grant and would like your assistance in evaluating several responses to a question pertaining to the abstract. Here is the abstract: *Paste in Abstract

**ENTER**

I am going to provide you with two different responses to a question about the grant abstract provided above. The question is: “Briefly summarize the primary outcome this research aims to investigate or achieve.” For each response, I'd like you to evaluate two primary metrics: Detail and Depth, and Relevance and Completeness. Please provide me with the evaluations (each response evaluated as a 1, 2, 3, 4, or 5) labeled in order with the number corresponding to the response I input.

**Detail and Depth:**

1 (Very Low): The response lacks detail and depth. It is extremely brief and provides minimal information or analysis.

2 (Low): The response has limited detail and depth. It offers some information, but the analysis is superficial, and key points may be missing.

3 (Moderate): The response includes a moderate level of detail and depth. It provides a decent amount of information, but the analysis could be more thorough, and some aspects may lack clarity.

4 (High): The response demonstrates a high level of detail and depth. It includes comprehensive information, and the analysis is thorough, offering insights beyond the obvious.

5 (Very High): The response is exceptional in detail and depth. It goes above and beyond expectations, providing a comprehensive and insightful analysis with a thorough exploration of all relevant aspects.

**Relevance and Completeness:**

1 (Very Low): The response is highly irrelevant and incomplete. It fails to address key aspects of the question, and important information is missing.

2 (Low): The response is somewhat relevant, but there are notable gaps in completeness. It addresses some aspects of the question, but crucial points may be omitted.

3 (Moderate): The response is moderately relevant and complete. It covers most of the essential aspects of the question, but there may be some minor omissions or areas that lack clarity.

4 (High): The response is highly relevant and complete. It addresses all key aspects of the question, providing a thorough and comprehensive answer with no significant omissions.

5 (Very High): The response is exceptionally relevant and complete. It goes above and beyond in addressing the question, leaving no room for improvement. It is well-organized, and all relevant points are covered in depth.

After evaluating each response, please organize the evaluations in the same order as the responses provided, labeling each evaluation with the corresponding number of the response for easy matching.

**ENTER**

This is the first response: *Paste in GPT Response

This is the second response: *Paste in Human Response

**ENTER**

**Q1: INNOVATION**

I will provide you with an abstract from an NIH funded grant and would like your assistance in evaluating several responses to a question pertaining to the abstract. Here is the abstract: *Paste in Abstract

**ENTER**

I am going to provide you with two different responses to a question about the grant abstract provided above. The question is: What primary innovation or advancement is being pursued or tested in this research project? For each response, I'd like you to evaluate two primary metrics: Detail and Depth, and Relevance and Completeness. Please provide me with the evaluations (each response evaluated as a 1, 2, 3, 4, or 5) labeled in order with the number corresponding to the response I input.

**Detail and Depth:**

1 (Very Low): The response lacks detail and depth. It is extremely brief and provides minimal information or analysis.

2 (Low): The response has limited detail and depth. It offers some information, but the analysis is superficial, and key points may be missing.

3 (Moderate): The response includes a moderate level of detail and depth. It provides a decent amount of information, but the analysis could be more thorough, and some aspects may lack clarity.

4 (High): The response demonstrates a high level of detail and depth. It includes comprehensive information, and the analysis is thorough, offering insights beyond the obvious.

5 (Very High): The response is exceptional in detail and depth. It goes above and beyond expectations, providing a comprehensive and insightful analysis with a thorough exploration of all relevant aspects.

**Relevance and Completeness:**

1 (Very Low): The response is highly irrelevant and incomplete. It fails to address key aspects of the question, and important information is missing.

2 (Low): The response is somewhat relevant, but there are notable gaps in completeness. It addresses some aspects of the question, but crucial points may be omitted.

3 (Moderate): The response is moderately relevant and complete. It covers most of the essential aspects of the question, but there may be some minor omissions or areas that lack clarity.

4 (High): The response is highly relevant and complete. It addresses all key aspects of the question, providing a thorough and comprehensive answer with no significant omissions.

5 (Very High): The response is exceptionally relevant and complete. It goes above and beyond in addressing the question, leaving no room for improvement. It is well-organized, and all relevant points are covered in depth.

After evaluating each response, please organize the evaluations in the same order as the responses provided, labeling each evaluation with the corresponding number of the response for easy matching.

**ENTER**

This is the first response: *Paste in GPT Response

This is the second response: *Paste in Human Response

**ENTER**
