# Supplementary material for: AI versus human coding of NIH grant abstracts
Source: PLoS One. 2026 Oct 8;21(10):e0343857. doi: 10.1371/journal.pone.0343857 (PMC13648982; doi:10.1371/journal.pone.0343857)
Supplement: Supplementary file 2 [file pone.0343857.s004.docx]

GPT Script: **I am going to provide you with different responses to this question: What primary innovation or advancement is being pursued or tested in this NIH research project? Suggest five discrete categories in which these studies could be placed that would help to classify the kinds of interventions, practices or topics being tested.**

| **GPT Categories** | **Human Categories** |
| --- | --- |
| **1. Medication-Assisted Treatment (MAT) and Pharmacotherapy Innovations:** This category includes research focused on evaluating the effectiveness and implementation of pharmacological treatments, such as buprenorphine, methadone, and naltrexone, in various settings. It encompasses studies comparing different forms of MAT, assessing the impact of extended-release formulations, and integrating MAT in community and criminal justice settings to improve access and outcomes for individuals with Opioid Use Disorder (OUD). | **1. Medication-Assisted Treatment (MAT) and Pharmacotherapy Advances:** This category encompasses research projects focused on the development, evaluation, and comparison of pharmacological treatments for opioid use disorder (OUD) and other substance use disorders (SUDs). Studies within this category aim to improve the efficacy, accessibility, and outcomes of treatments like buprenorphine (including extended-release formulations), naltrexone, and other medication-assisted treatments within various settings, including correctional facilities, primary care, and emergency departments. |
| **2. Technology and Data Analytics in Public Health and Treatment:** Projects under this category leverage technology, including mobile health (mHealth), web-based platforms, and data analytics (like natural language processing and machine learning), to improve screening, treatment delivery, and monitoring of substance use disorders. Innovations include developing online training programs for clinicians, telemedicine-based interventions, and platforms for analyzing social "big data" for public health surveillance. | **2. Integration of SUD Treatment in Healthcare Settings:** Research projects under this classification are aimed at integrating SUD treatment into broader healthcare and community settings. This includes leveraging existing healthcare infrastructure (such as primary care and emergency departments) to address SUDs, hepatitis C, HIV, and opioid overdose through integrated care models. The goal is to enhance the identification, engagement, and treatment of individuals with SUDs within medical settings and the community. |
| **3. Integration of Substance Use Disorder Treatment into Healthcare and Community Settings:** This area focuses on integrating SUD treatment and harm reduction strategies into primary care, emergency departments, and community settings. It includes implementing screening and brief interventions, enhancing addiction treatment within healthcare systems through various models of care, and establishing recovery support services outside of clinical environments. | **3. Digital and Mobile Health Innovations:** This category includes projects that utilize technology to address SUDs and related public health concerns. Innovations range from automated platforms for analyzing social media data to predict HIV incidence, to mobile health interventions for improving treatment retention and adherence. Also included are efforts to use digital tools for public health surveillance, such as identifying emerging drug trends through the National Early Warning System (NEWS). |
| **4. Innovations in Harm Reduction and Overdose Prevention:** Projects in this category aim to develop and evaluate interventions to reduce the harm associated with drug use, particularly opioid and polysubstance use. This includes evaluating naloxone distribution and education programs, developing harm reduction services tailored to specific populations, and creating interventions to prevent overdose deaths in communities. | **4. Harm Reduction and Overdose Prevention Strategies:** Projects in this category focus on developing, testing, and implementing harm reduction strategies aimed at reducing the harms associated with drug use, including overdose prevention. Innovations include naloxone distribution and education, mobile harm reduction services tailored to specific populations (such as women or rural communities), and strategies to improve access to and engagement with harm reduction services. |
| **5. Behavioral and Psychosocial Interventions:** Research projects that fall into this category are focused on evaluating and improving non-pharmacological interventions for substance use disorders. This includes cognitive-behavioral therapies, mindfulness-based interventions, peer support models, and family-based recovery support services. These studies aim to address the psychological, social, and behavioral aspects of addiction and recovery. | **5. Behavioral, Social, and Community-Based Interventions:** This category encompasses research aimed at enhancing behavioral, social, and community-based interventions for individuals with SUDs. Projects focus on a wide range of interventions, including cognitive-behavioral therapies, mindfulness-based interventions, peer support models, and family-based recovery support services. The goal is to address the psychological, social, and environmental factors that contribute to SUDs and support recovery. |
